# Mechanical characterization of human brain tumors from patients and comparison to potential surgical phantoms

**DOI:** 10.1371/journal.pone.0177561

**Published:** 2017-06-05

**Authors:** Daniel C. Stewart, Andrés Rubiano, Kyle Dyson, Chelsey S. Simmons

**Affiliations:** 1 J. Crayton Pruitt Family Department of Biomedical Engineering, University of Florida, Gainesville, Florida, United States of America; 2 Department of Mechanical and Aerospace Engineering, University of Florida, Gainesville, Florida, United States of America; 3 Lillian S. Wells Department of Neurosurgery, University of Florida, Gainesville, Florida, United States of America; 4 Division of Cardiovascular Medicine, University of Florida, Gainesville, Florida, United States of America; University of California, San Diego, UNITED STATES

## Abstract

While mechanical properties of the brain have been investigated thoroughly, the mechanical properties of human brain tumors rarely have been directly quantified due to the complexities of acquiring human tissue. Quantifying the mechanical properties of brain tumors is a necessary prerequisite, though, to identify appropriate materials for surgical tool testing and to define target parameters for cell biology and tissue engineering applications. Since characterization methods vary widely for soft biological and synthetic materials, here, we have developed a characterization method compatible with abnormally shaped human brain tumors, mouse tumors, animal tissue and common hydrogels, which enables direct comparison among samples. Samples were tested using a custom-built millimeter-scale indenter, and resulting force-displacement data is analyzed to quantify the steady-state modulus of each sample. We have directly quantified the quasi-static mechanical properties of human brain tumors with effective moduli ranging from 0.17–16.06 kPa for various pathologies. Of the readily available and inexpensive animal tissues tested, chicken liver (steady-state modulus 0.44 ± 0.13 kPa) has similar mechanical properties to normal human brain tissue while chicken crassus gizzard muscle (steady-state modulus 3.00 ± 0.65 kPa) has similar mechanical properties to human brain tumors. Other materials frequently used to mimic brain tissue in mechanical tests, like ballistic gel and chicken breast, were found to be significantly stiffer than both normal and diseased brain tissue. We have directly compared quasi-static properties of brain tissue, brain tumors, and common mechanical surrogates, though additional tests would be required to determine more complex constitutive models.

## Introduction

Freshly isolated human tissue samples are complicated to procure for a wide range of logistical and regulatory reasons [1,2]. Consequently, researchers rely on animal models and hydrogels to mimic the mechanical behavior of the brain for a variety of applications including high strain rate trauma [3–7], neurosurgical procedures [8,9], and mechanobiology experiments [10]. Hydrogel surrogates are particularly useful for surgical training, medical device development, and mechanical characterization given their customizable fabrication, transparent nature for visualization, and ability to incorporate fiducial markers for deformation tracking. While bovine and porcine brains are relatively easy to obtain and have been used historically for brain mechanics simulations [11–13], normal animal brain tissues may not be appropriate to mimic brain tumors that have dramatically different cell, matrix, and vascular composition than normal tissue.

**Fig 1:**
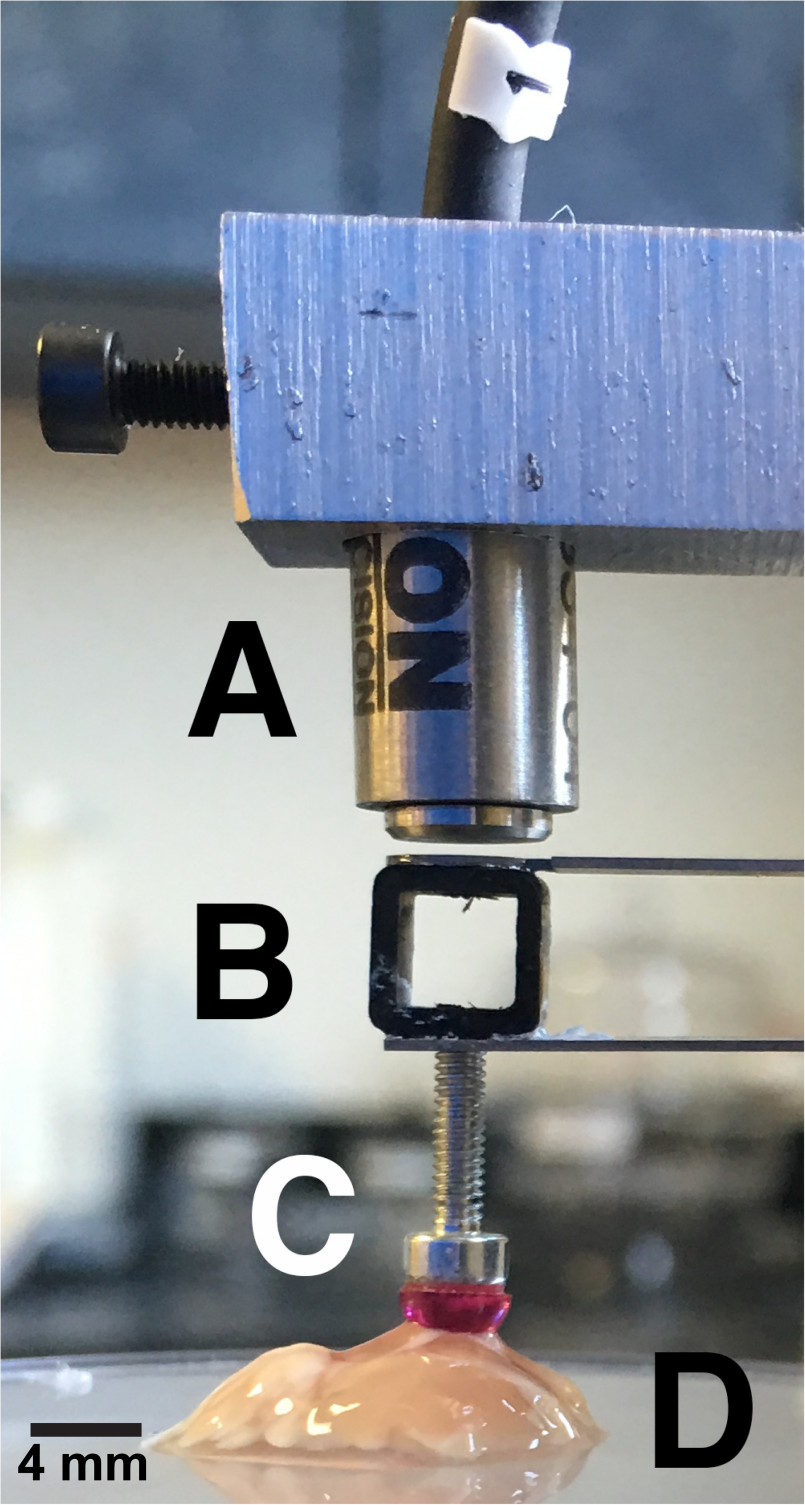
Multi-scale indenter can quantify quasi-static properties of arbitrarily shaped soft matter and biological tissue samples. A capacitive probe (A) measures capacitive changes due to the deflection of a titanium cantilever (B) as a sample is indented. A 4 mm-diameter ruby tip (C) at the free end of the cantilever comes into contact with the sample (D). The image depicts a posterior coronal cross-section of a mouse brain sample being indented. Capacitive probe is able to measure deflections on the order of 10 nm, allowing for quantification of μN-level forces.

As with most human tissue, human brain tumors of adequate size for mechanical testing are difficult to obtain, though, as access to patients is limited and portions of excised tumors must also be retained for clinical and diagnostic uses [14]. Furthermore, excised tumors rapidly degrade once excised from the *in situ* environment, are of arbitrary size and geometry, and possess large intra- and inter-sample heterogeneity. Taken together, these factors constrain options for mechanical characterization of human brain tumors. We have built a cantilever-based multi-scale indenter (MSI, Fig 1) that allows us to test tissue-level mechanics and determine the mechanical properties of small, arbitrarily shaped samples [15,16].

While effective mechanical properties of brain tumors have been estimated using non-invasive methods [17], bulk, tissue-level mechanical properties of freshly isolated human brain tumors have only recently been investigated. Miroshnikova et al. performed microscale indentation of fresh and frozen low-grade-glioma, primary glioblastoma, and non-tumor gliosis human biopsy samples using AFM [18]. However, mechanical properties of common surrogates for normal brain tissue like ballistic gelatin [19,20] and agarose [21,22] have not been directly compared to brain tumor mechanical properties using identical equipment and methods. Since breast tumors [23] and pancreatic tumors [24] were found to be 5–10 times stiffer than the surrounding normal tissue, we anticipate brain tumors would also have different properties than neighboring normal tissue. Furthermore, different types of brain tumors (e.g. glioblastoma, meningioma, astrocytoma) each have different cell- and tissue-level properties which suggests different types of tumors may have different properties. Meat prepared for consumption could provide an inexpensive and simple source of surrogate tissue for medical device testing and surgical planning; however, the mechanical properties of such samples have not been directly compared to the human tissue or tumors for which they may substitute.

Here, we quantify quasi-static mechanical properties of human brain tumors, mouse brain tumors, and consumer products using identical equipment, models, and analysis for direct comparison.

## Materials and methods

### Preparation of tissue and surrogate samples

Human brain tumors were collected post-surgery from the Florida Center for Brain Tumor Research (FCBTR) under University of Florida (UF) Institutional Review Board-approved protocol IRB201500268. This study was exempt as samples and data were analyzed anonymously after patients gave written informed consent for collection under IRB#134–2006. Samples were placed in culture media and on ice during transport and between indentations. Collected samples were sliced to approximately 3 mm thick with a matrix slicer (Zivic Instruments) and indented within 3–4 hours after isolation. Excised tumors ranged between 20–150 mg with volumes approximately 1 cm^3^. While the thickness of the sample can be controlled using matrix slicers, overall morphology of clinically excised tumors varies wildly. Our MSI and subsequent analysis (see Results and Discussion) was designed to test samples of arbitrary geometries as long as the thickness of the sample is controlled. Samples were kept hydrated in between indentations by rinsing them in saline and kept in a pool of saline during indentation.

A mouse model was used to compare matched normal brain and tumors under UF Institutional Animal Care and Use Committee-approved number 201607966. The astrocytoma tumor cell line, KR158B-luc, was isolated from a spontaneously arising tumor in an Nf1;Trp53 mutant mouse on a C57BL/6 background [25] and cultured under normal conditions. Tumor implantation was performed as previously described under isoflurane anesthesia [26]. Briefly, 20 x 10^3^ KR158B cells in equal parts Dulbecco’s phosphate-buffered saline (DPBS) and 10% methyl-cellulose (R&D Systems) were injected into 5–8 week old C57BL/6 mice. Tumor-bearing mice were euthanized using CO2 inhalation at pre-approved humane endpoints 25–35 days after implantation.

Samples of chicken tissues were purchased from a local grocery store that prepared meat within 1–2 days after butchering, per USDA guidelines. After purchase, samples were stored in packaging from the butcher at 4°C for no more than 2 days and indented within 4 hours after being cut from the whole tissue. Large portions of each chicken surrogate (breast, liver, and the tenuis and crassus muscle of the gizzard) were taken from four different samples. Each portion was then placed in a matrix slicer (Zivic Instruments) to control the thickness of the sample; the breast, liver, and crassus muscle were cut to be 3 mm thick for easy handling, while tenuis muscle samples, due to their natural thin dimensions, were cut to be 1 mm thick.

Three different concentrations of agarose solution, 0.2%, 0.4%, and 0.6% agarose (UltraPure^TM^ Agarose, Life Technologies Corporation), were prepared per manufacturer’s instructions. Ballistic gel samples (Clear Ballistics LLC) were purchased pre-solidified and melted per manufacturer’s instructions. Knox® gelatin was purchased from a local grocery store and samples made per manufacturer’s instructions. All gel solutions were solidified in glass petri dishes to control sample thickness at ~3 mm; the flat center region of the petri dish was indented between 10–16 times per material.

Collagen hydrogels were assembled from a solution of 5x DMEM in HEPES (Sigma Aldrich and Gibco, respectively) and rat tail collagen type-I (Corning) diluted with 0.2% glacial acetic acid (Fisher Scientific) in PBS to obtain 2, 3, and 4 mg/mL final collagen concentration. A 55 μL aliquot of collagen solution was added to individual wells of a 96-well tissue culture plate and placed in a 37^°^C incubator for 35 minutes to allow thermogelling. Samples were either stored at 4^°^C submerged in PBS or treated with 120μL of a 1% gluteraldehyde solution (Sigma Aldrich) in PBS for 2 or 4 hours before being rinsed and stored in PBS. All gels were indented 4 hours after fabrication with storage in PBS or glutaraldehyde as indicated.

### Multi-scale indenter and relaxation tests

A custom cantilever-based indenter (Fig 1) [16] was used to indent tissue samples and record force relaxation over time. A piezoelectric stage (P-628.1CD, Physik Instrumente) displaced a soft titanium cantilever with a 4 mm-diameter rigid tip. Cantilever stiffness (79.8 N/m) was calibrated directly with small weights hung from the cantilever tip. A custom program in LabVIEW (National Instruments) was used to control indentation profile and to read deflection of cantilever tip with capacitive sensor (C8S-3.2–2.0 and compact driver CD1-CD6, Lion Precision) through a data acquisition card system (NI 9220 and cDAQ-9171, National Instruments). The cantilever base was driven to 10% of the total thickness of the samples at 15 *μ*m/s. The cantilever was then held as this position while the tissue underwent stress relaxation. Stress relaxation times varied between 60–180 s to allow the various samples to reach a quasi-static state. Samples were kept hydrated in between indentations by submerging them in 1x DMEM cell media (Dow Corning).

### Adjusted Hertz model for determination of effective modulus

Our custom indentation system was designed specifically to measure mechanical properties of biological and soft matter. By measuring the nanometer-scale displacement of the tip into a sample with the capacitive probe and knowing the stiffness of the titanium cantilever, we can calculate the small, *μ*N-scale forces between the tip and the soft sample. Hertz contact theory is frequently used in mechanical characterization studies to correlate force as a function of indentation depth, thereby identifying an elastic modulus, E_Hertz_:

$$F\left(t\right)=\frac{4E_{\mathrm{Hertz}}\cdot \sqrt{R}\cdot \delta {\left(t\right)}^{\frac{3}{2}}}{3\left(1-\nu^{2}\right)}$$ (1)

where *F* = force calculated by calibrated cantilever stiffness and displacement, *R* = radius of the sphere in contact with the surface, *δ* = the indentation depth, and *ν* = Poisson’s ratio. This formulation is often used to characterize soft matter with indentation [27,28].

The Hertz contact model hypothetically is useful in characterization of soft matter as it can readily be applied to samples of arbitrary shape as long as a flat surface is present [16,29–31]. However, biological and soft materials violate the core assumptions of the model Hertz developed in the late 1800’s for materials that are flat, linearly elastic, isotropic, and homogeneous [32–34]. Soft tissues such as the brain obviously violate these assumptions as brain tissues and tumors exhibit high viscoelastic behavior, anisotropy, and heterogeneity due to the varying cellular-level compositions. We can mitigate some of these limitations in our indentation procedures by controlling the parameters on our MSI, but ultimately the Hertz model can only yield an effective modulus as a helpful relative value for soft materials that so dramatically violate its assumptions. We operate at the millimeter scale with our indentation tip (Fig 1) so that the tissue is more homogeneous than the nano/micrometer-scale captured by AFM, for example. In addition, using a matrix slicer to cut tissue samples attempts at a more even, flat surface for better characterization of tissue-level properties. Our capacitive probe, which is able to detect nm-level deflections, allows us to constrain to small deformations and remain in a somewhat linear region. Hertz also assumes that there is no friction or adhesion between the two surfaces, which we reduce by keeping samples hydrated with culture media, using an optically polished ruby tip to indent the tissue, and constraining our analysis to the relaxation phase of indentation where adhesion is less of a concern.

Despite the material-related limitations of utilizing the Hertz contact model for biological applications, our MSI is sensitive and precise enough for control of the dimensional parameters also assumed in Hertz’s original derivation [33]. Hertz commented that the indentation depth should be less than 10% of the diameter of the contact area and thus ~1% of the diameter of the spherical indenter [33]; however, this constraint is often operationalized in the literature to be less than 10% of the indenter diameter [27]. Experimentally, we have found that our steady-state modulus described below is consistent for indentation depths ~10% of the indenter diameter and that this depth also allows for more robust data acquisition at higher detectable forces, so we also have used this less restrictive interpretation.

**Fig 2:**
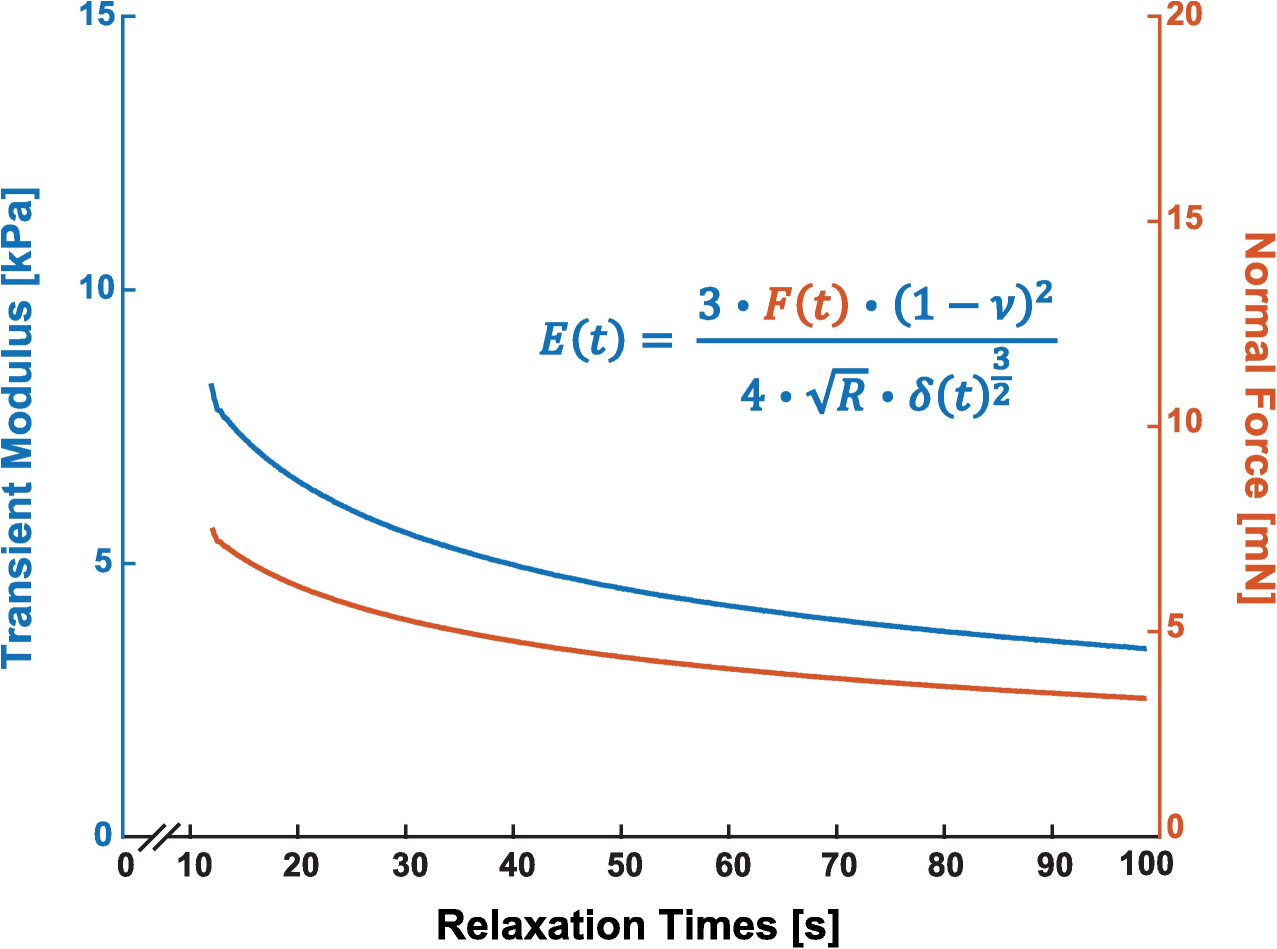
Effective modulus of a 0.4% agarose gel sample is determined by a time-dependent rearrangement of the Hertz contact model. Force displacement data (lower orange curve) is converted to the effective modulus (upper blue curve) using the Hertz contact model for a sphere and a half space (Eq 2, inset). Samples were indented at a rate of 15 *μ*m/s followed by a stress-relaxation phase where the base of the cantilever is held at a constant strain until the tissue fully relaxes.

To determine the effective modulus of a soft sample in a quasi-static state, we rearranged the Hertz contact model for a parabolic contact area to determine an effective modulus as a function of time:

$$E_{\mathrm{effective}}\left(t\right)=\frac{3\cdot F\left(t\right)\cdot \left(1-\nu^{2}\right)}{4\cdot \sqrt{R}\cdot \delta {\left(t\right)}^{\frac{3}{2}}}$$ (2)

where *F* = force as calculated by tip displacement multiplied by calibrated stiffness; *R* = radius of spherical indenter tip; *δ* = indentation depth calculated as stage movement minus deflection of tip; and *ν* = Poisson’s Ratio (Fig 2). This rearrangement is comparable to a viscoelastic stress-relaxation modulus that considers instantaneous changes in a total effective modulus during stress-relaxation experiments [35]. In our experiments and analyses, *R* = 2 mm, *δ*_*max*_ ≈ 300 μm for most samples, and *ν* = 0.4. In previous mechanical characterization studies of the brain, a Poisson’s ratio between 0.4 and 0.496 has been used [36]. While incompressibility is often assumed for tissue, we have measured values ranging from *ν* ≈ 0.35–0.4 for biological tissues in our lab (chicken liver, this work; rat heart) [16]. Variations in Poisson’s ratio ± 0.05 affect values of calculated SSM less than 10%, so we did not attempt to carefully quantify Poisson’s ratio for each material.

### Determination of steady-state modulus and characteristic time

**Fig 3:**
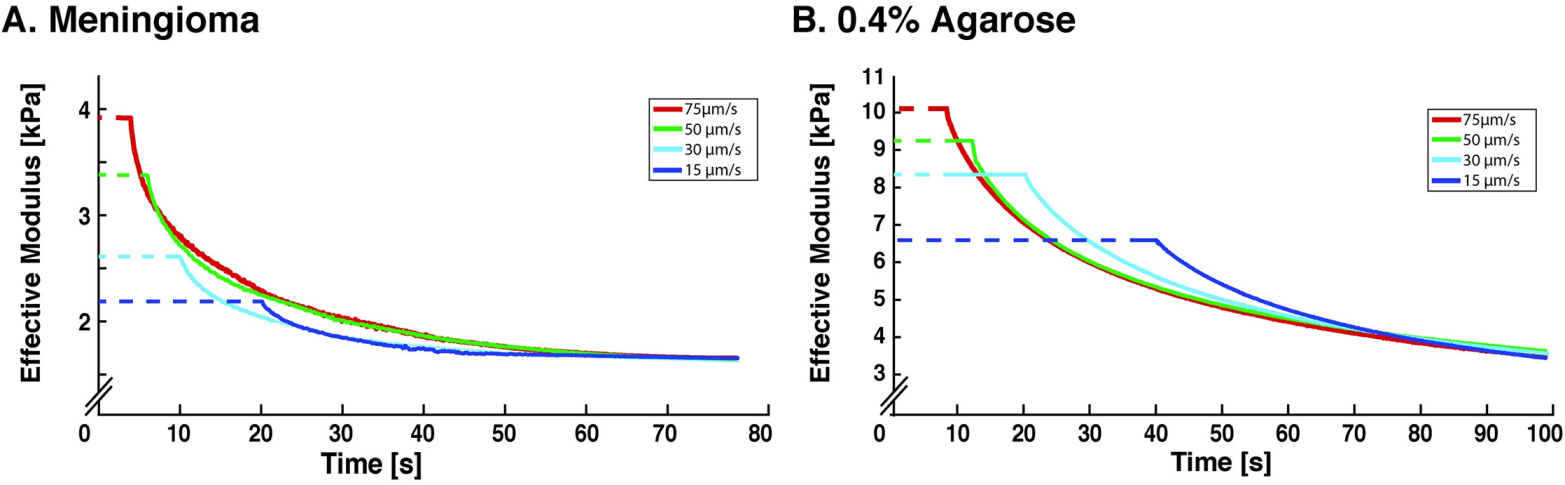
Steady-state mechanical properties are independent of initial strain rate. Samples of (A) meningioma and (B) 0.4% agarose hydrogel were indented with our MSI to 10% of sample thickness (3 mm sample thickness, 300 μm indentation depth) at 15, 30, 50, and 75 *μ*m/s and subsequently allowed to undergo stress relaxation. Given adequate relaxation time, samples relaxed and converged to a similar steady-state modulus. This robust quasi-static property of tissues allows us to use the steady-state modulus as a simple metric for direct comparison of brain tissue and hydrogels. N.B. Scales of the effective modulus axes are different to allow clear visualization of stress-relaxation behavior.

In our experiments, relaxation times varied between surrogates; stiffer materials needed longer relaxation time. However, consistently across all surrogates tested, forces and thus effective modulus reached a steady-state independent of the initial indentation rate (Fig 3). We thus chose to name the effective modulus at this equilibrium state the Steady-State Modulus (SSM), similar to a stress-relaxation modulus at t = *∞* [37]. Relaxation was not observed when indenting crosslinked silicone in our system, which supports SSM as a meaningful quantity to describe relaxation of viscoelastic (or poroelastic) materials (S1 Fig).

We chose the Standard Linear Solid (SLS) to estimate a reasonable SSM. A typical spring-dashpot model for the viscoelastic SLS contains a single spring, *E*_*p*_, in parallel to a spring and dashpot in a series [37–39]. The single spring element is a reasonable representation of SSM, as the element can be considered independent of time unlike the other two elements. Thus, the SLS provides a simple model to which to fit our data to obtain SSM independent of time-dependent effects. The following equation represents the total effective behavior of a material with the SLS model:

$$E_{\mathrm{effective}}\left(t\right)=E_{p}+E_{s}e^{-\frac{t}{\tau }}$$ (3)

where *E*_*effective*_ is the effective, time-dependent modulus, *E*_*p*_ is the steady-state modulus after relaxation, and τ is the characteristic time equal to the quotient **η/**E_S_, where **η** is the viscosity and E_S_ is the instantaneous, strain-rate dependent modulus **[39]**. The characteristic time represents the viscous relaxation the tissue exhibits in response to a constant applied strain.

We fit Eq 3 to the relaxation phase of our indentations using the MATLAB Curve Fitting Toolbox (MathWorks) to calculate our SSM (*E*_*p*_). Fits were evaluated using the normalized mean-squared error (NMSE) metric determined by the Goodness of Fit function in MATLAB. Fits that had an NMSE below 0.4 were rejected, though most were above 0.8. Example fits are demonstrated in S2 Fig.

## Results and discussion

### Human brain tumors are stiffer than normal mouse brain

**Fig 4:**
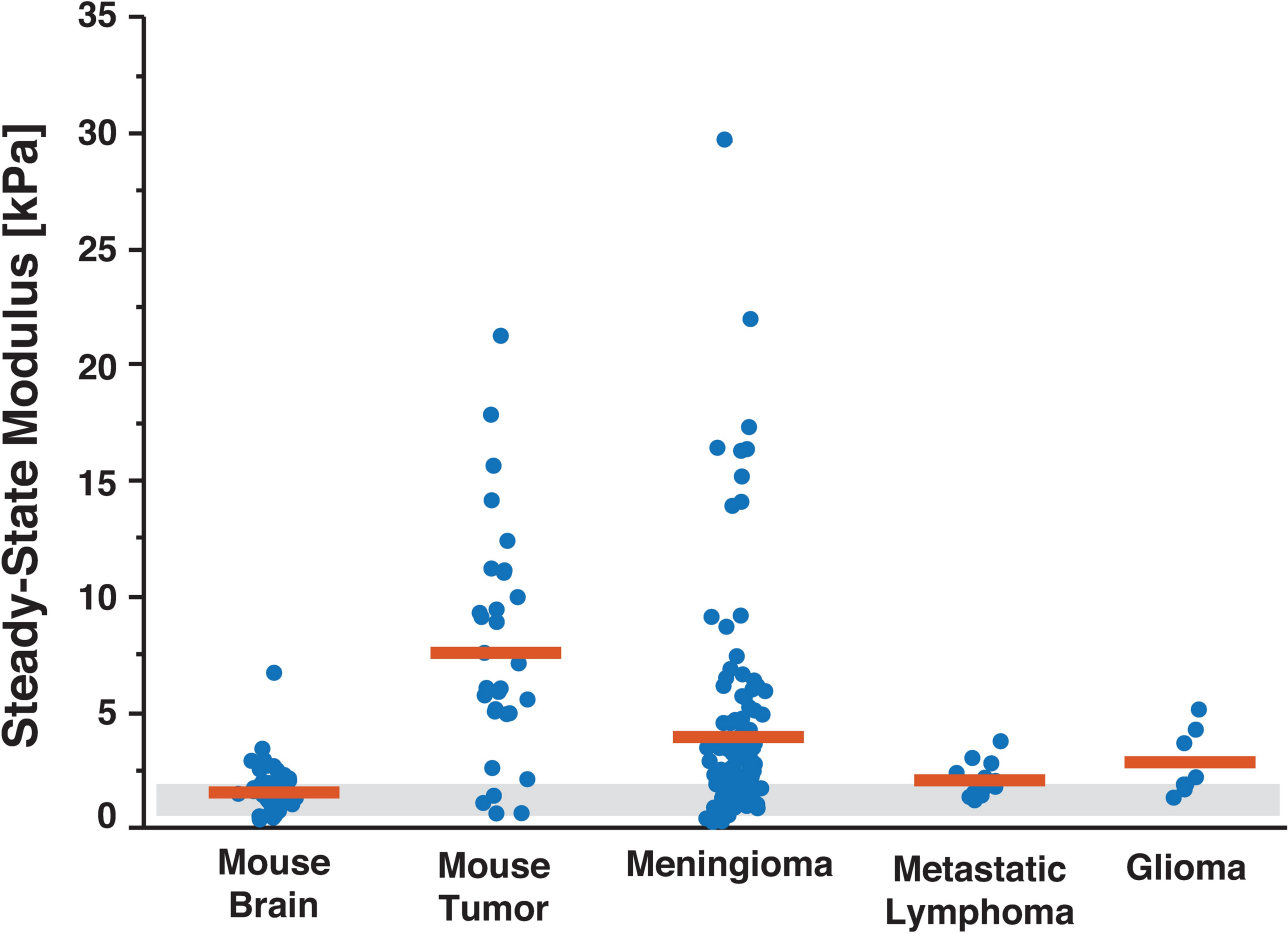
Human brain tumors are approximately twice as stiff as normal brain tissue. Mean (red bar) of pooled indentations show glioma (n = 8 indentations from 1 sample), meningioma (n = 118 indentations from 18 samples), and mouse tumors implanted in mice (n = 31 indentations from 9 samples) are stiffer and more heterogeneous than normal mouse brain tissue (n = 50 from 11 samples) tested in our lab and as previously reported in literature (grey box). In contrast, metastatic lymphomas (n = 14 indentations from 3 samples from one patient) showed no significant difference between the SSM of normal brain tissue and were not as stiff as other tumors. Grey box reflects values for normal brain modulus reported in the literature using unconfined compression [40] and indentation methods [7,41–43].

We have directly quantified mechanical properties of freshly isolated human brain tumors. Using our SSM parameter, we compared the effective modulus of diseased brain tissue (gliomas, meningiomas, metastatic lymphomas, and mouse tumors grown in mice) to normal mouse brain tissue (Fig 4). Human meningiomas demonstrated a higher SSM than freshly-isolated mouse brain (3.97 ± 3.66 kPa to 1.56 ± 0.75 kPa, respectively [mean ± SD]), as did mouse tumors (7.64 ± 4.73 kPa). Statistical significance for multiple comparisons using non-parametric Wilcoxon test (JMP analysis software by SAS) is reported in Table 1. Human glioma (2.75 ± 1.40 kPa) and metastatic lymphoma (2.10 ± 0.57 kPa) were more similar to normal brain tissue (Fig 4).

**Table 1:** Results of multiple comparisons across relevant surrogates tested.

*Wilcoxon p-values for Steady-State Modulus*		Human	Mouse		Chicken				Agarose			Gelatin
		Menin^a^	Tumor	Brain	Liver	Crassus	Tenuis	Breast	0.2%	0.4%	0.6%	Knox®
**Human**	Menin^a^		0.0168	0.0006	0.0043	**0.0969**	0.0043	0.0073	**0.7191**	0.0005	<0.0001	0.0192
**Mouse**	Tumor	0.0168		0.0030	0.0372	**0.1427**	0.0253	0.0372	0.0048	**0.8383**	0.0024	**0.8170**
	Brain	0.0006	0.0030		**0.4727**	0.0050	0.0050	0.0050	0.0004	0.0001	<0.0001	0.0050
**Chicken**	Liver	0.0043	0.0372	**0.4727**		0.0304	0.0304	0.0304	0.0058	0.0058	0.0032	0.0304
	Crassus	**0.0969**	**0.1427**	0.0050	0.0304		0.0304	0.0304	0.0058	0.0196	0.0032	0.0304
	Tenuis	0.0043	0.0253	0.0050	0.0304	0.0304		0.6650	0.0058	0.0133	0.7263	0.0606
	Breast	0.0073	0.0372	0.0050	0.0304	0.0304	0.6650		0.0058	0.0058	0.9601	0.0304
**Agarose**	0.2%	**0.7191**	0.0048	0.0004	0.0058	0.0058	0.0058	0.0058		0.0002	<0.001	0.0058
	0.4%	0.0005	**0.8383**	0.0001	0.0058	0.0196	0.0133	0.0058	0.0002		<0.001	0.9436
	0.6%	<0.0001	0.0024	<0.0001	0.0032	0.0032	0.7263	0.9601	<0.001	<0.001		0.0032
**Gelatin**	Knox®	0.0192	**0.8170**	0.0050	0.0304	0.0304	0.0606	0.0304	0.0058	0.9436	0.0032	

P-values were obtained by performing a non-parametric Wilcoxon test for multiple comparisons in JMP software (SAS).

Grey boxes indicate tissues/surrogates that are *not* statistically different (p > 0.05), and values in bold indicate potential surrogates for normal brain and brain tumors in quasi-static in vitro applications.

^a^Menin = human meningioma

Our findings of brain tumor samples having >2-fold increase in modulus compared to healthy tissue are similar to a previous study using ultrasound elastography methods [17] and AFM [18]. Chauvet et al. used shear wave elastography to quantify the elastic properties of normal and diseased brain and found that the stiffness ranged from 11.4–33.1 kPa for various grades of brain tumors compared to a stiffness of 7.3 kPa for normal brain [17]. Elastography yields relative values, though, and cannot be compared easily to ex vivo hydrogels [44]. Furthermore, increased tumor stiffness compared to surrounding tissue is reasonable for certain pathologies since tumor matrices often contain a more abundant and tightly-packed cellular network, and tumor stiffening has been seen in mammary and pancreatic tissues [23,24].

Our custom MSI and analytical methods allow us to characterize millimeter-scale soft samples with abnormal geometry precisely enough to detect significant differences in SSM among tissues. While complex, constitutive models are useful for analyzing dynamic mechanical behavior of the brain, the SSM quasi-static metric provides a useful baseline for researchers seeking to mimic organ systems in surgical training, prepare mechanically biomimetic cell and tissue culture substrates, evaluate new medical devices and, importantly, compare mechanics-related findings across experiments and research groups.

**Fig 5:**
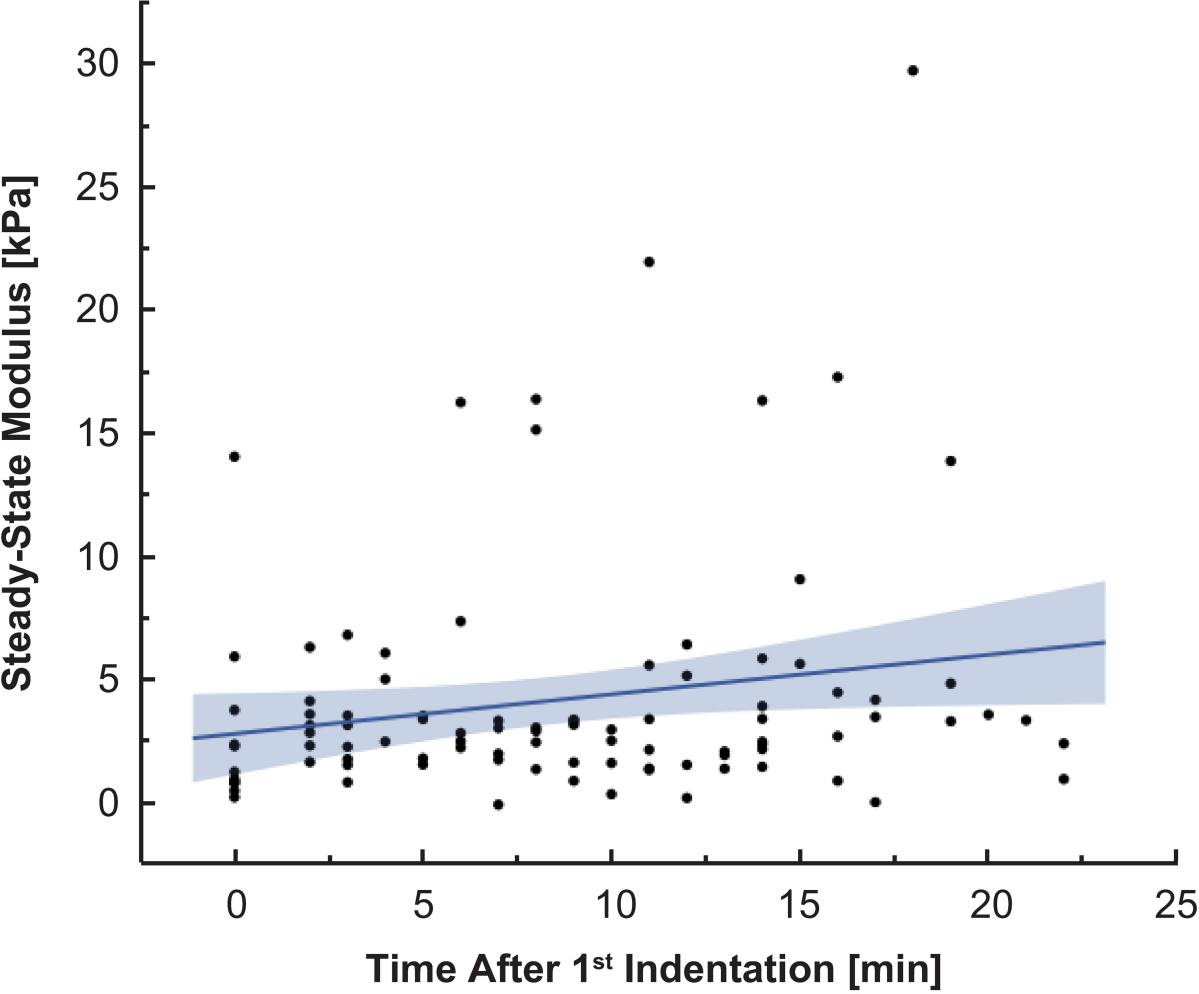
Calculated steady-state modulus showed no real change across multiple indentations over time on the same sample. 11 meningioma samples are plotted that were indented at least 6 times over the course of 30 min. The resulting steady-state modulus over each indentation and across multiple samples show no meaningful correlation (R^2^ = 0.04), indicating that no stiffening occurred as the sample was being tested and thus potential issues like dehydration and cell death are minimal. This reinforces that spread shown in calculated modulus values reflect actual differences in the mechanical properties of the tissue and not testing procedures.

As is evidenced in the data variability, tumors exhibit high levels of intra- and inter-sample heterogeneity. By performing our indentations at the millimeter-scale, we are reducing the variation that would be captured with micro/nanoscale indentations. In samples that were indented at least six times, no significant variation in calculated SSM was noticed between indentations, indicating that experimental conditions did not affect measurements appreciably (Fig 5). Means were calculated by averaging the mean SSM of multiple indentations for each sample tested to compensate for non-uniform geometries.

### Common mechanical hydrogel surrogates are stiffer than normal brain tissue

**Fig 6:**
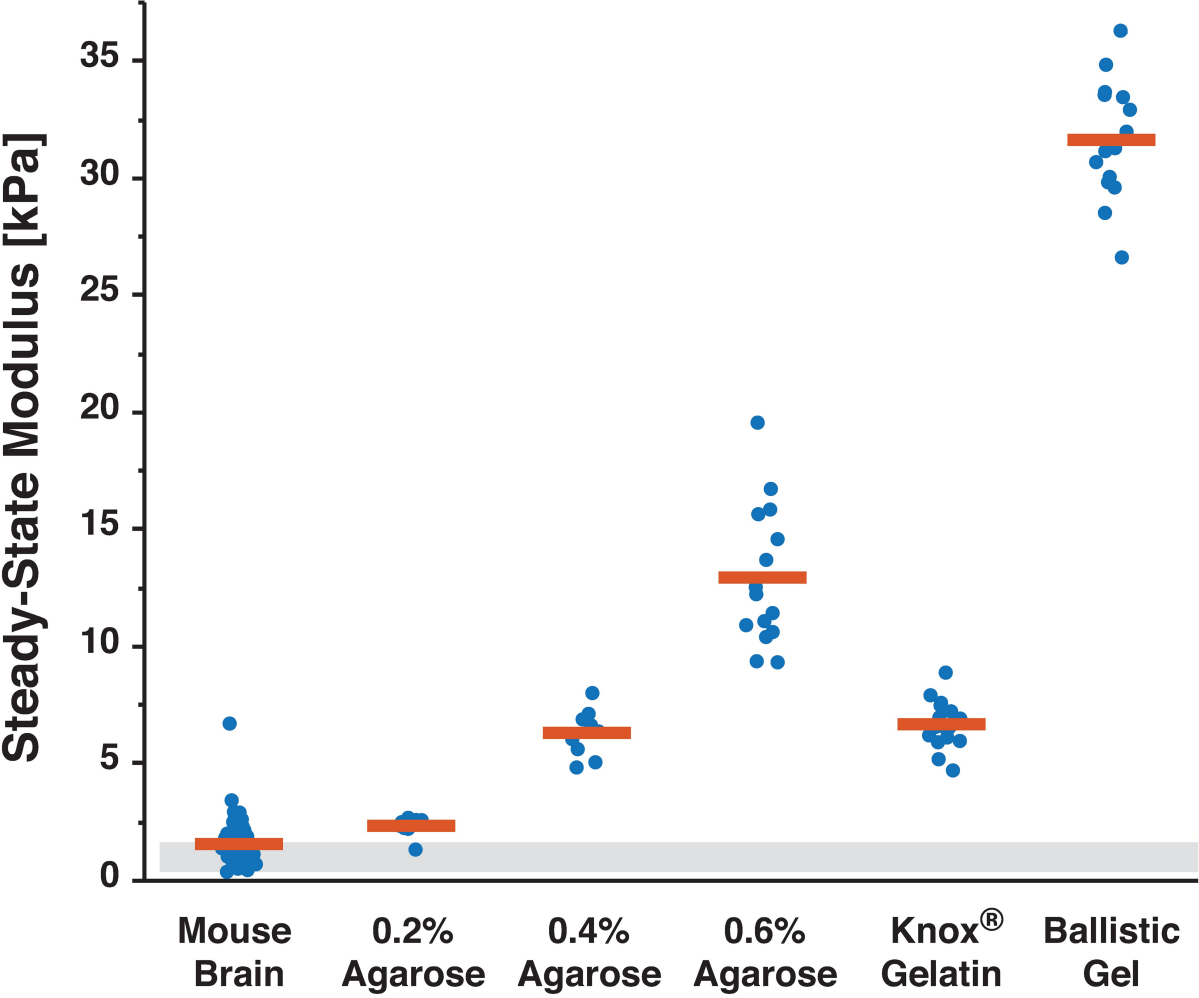
Agarose hydrogels and ballistic gel are stiffer than normal brain tissue. Mean (red bar) of pooled indentations are shown along with resulting steady-state modulus from each indentation performed. Despite being used frequently as mechanical surrogates for healthy brain tissue, low concentration agarose samples and a ballistic gel were stiffer than tested normal mouse brain samples. Store-bought Knox® Gelatin samples were also stiffer than normal brain tissue yet similar to the agarose hydrogels tested. Grey box reflects values for normal brain modulus reported in the literature using unconfined compression [40] and indentation methods [41,42].

Many common laboratory gel surrogates demonstrated higher steady-state moduli than normal brain tissue (Fig 6). Knox® gelatin samples (6.68 ± 0.49 kPa) were significantly stiffer than normal mouse brain tissue (p = 0.005), as was the softest hydrogel tested, 0.2% agarose (SSM = 2.35 ± 0.39 kPa, p = 0.0004, Table 1). Agarose (0.2%) was similar instead to meningiomas (p = 0.7191). As the concentration increased to 0.4% and 0.6% agarose, the SSM increased to 6.31 ± 0.96 kPa and 12.93 ± 2.99 kPa, respectively. While these concentrations previously have been reported to mimic normal brain tissue for mechanics experiments [21,22,45], agarose hydrogels actually have steady-state moduli significantly higher than normal brain tissue and in fact similar to brain tumors (Table 1). We investigated whether lowering the concentration of agarose would decrease the SSM to a range soft enough to mechanically mimic brain; however, even the lowest concentration of agarose tested (2%) was still significantly higher than normal mouse brain tissue (p<0.0004, Table 1).

While agarose is a common choice for mimicking the mechanical properties of brain for surgical tools and shock tubes, many other hydrogels are used to mimic brain mechanics for cell culture. Polyacrylamide and polydimethylsiloxane (PDMS) are often used for 2D studies, while polyethylene-glycol (PEG) and collagen are often used to encapsulate cells in 3D (see exemplary review by Lin et al[46]). Collagen hydrogels have been used readily for nervous tissue scaffolds and neural tissue engineering[47–51], and a recent example from Wang et al. demonstrates the utility of biomimetic hydrogels to study the effects of ECM stiffness on glioblastoma cells proliferation and subsequent remodeling of the 3D hydrogel [52]. Since different characterization methods can obtain different mechanical properties (see example discussion regarding gelatin in Bettadapur et al. [53]), we have included data on our own modified collagen gels for consistency (S3 Fig).

### Commercially available tissues can reflect range of normal and diseased brain tissue

**Fig 7:**
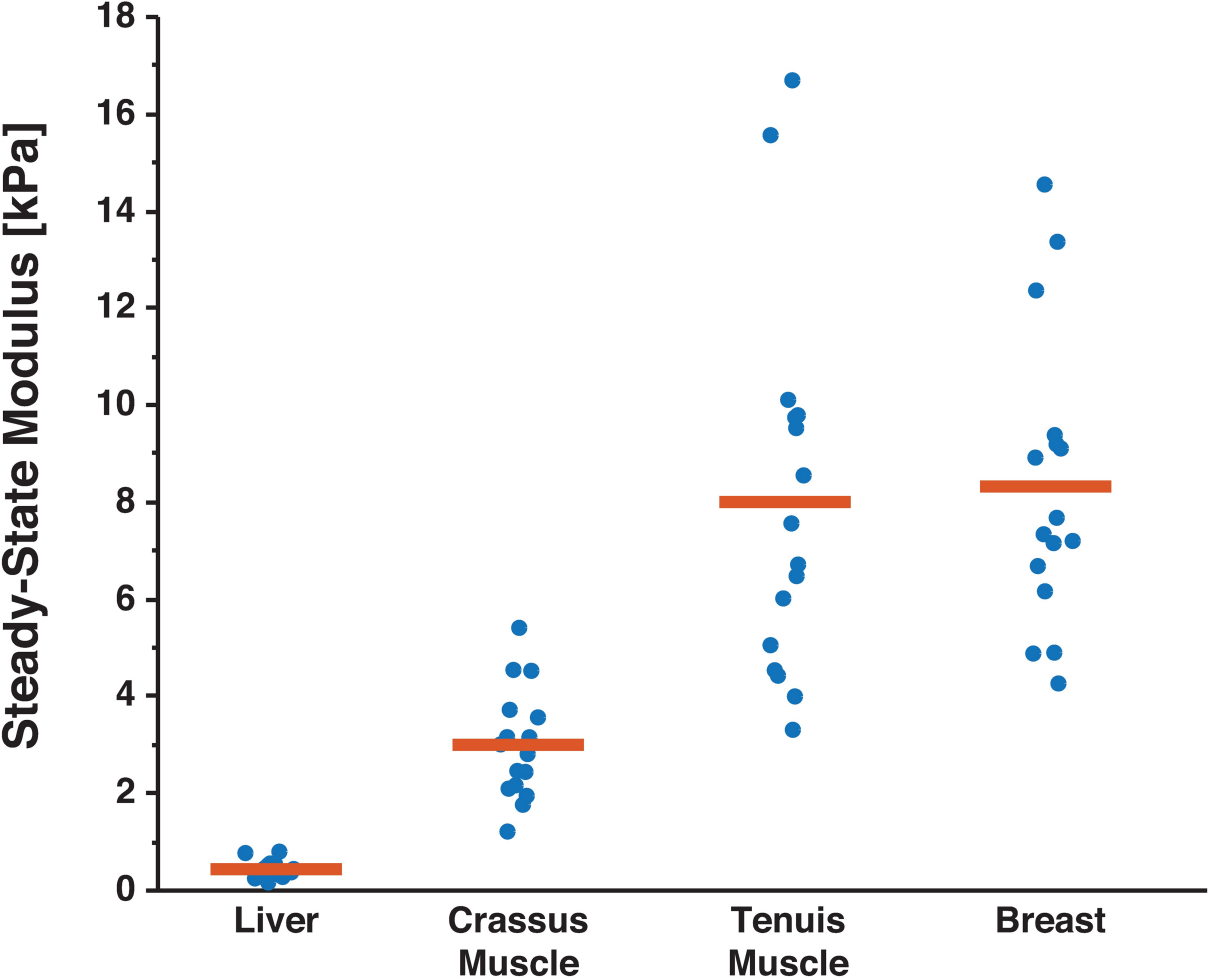
Readily-available store-bought meats are reasonable mechanical surrogates of soft tissues. Chicken breast, liver, and tenuis and crassus muscle of the gizzard were indented to determine the steady-state properties (n = 4 indentations of 4 samples for each tissue type). Mean (red bar) of pooled indentations are shown along with resulting steady-state modulus from each indentation performed. Chicken breast and tenuis muscle of the gizzard had significantly higher steady-state moduli than the mouse brain and human brain tumors (see Fig 4 and Table 1), compared to the liver and crassus muscle of the gizzard. The liver and crassus muscle showed similar mechanical properties to normal brain tissue and human brain tumor samples, respectively. Given the low cost and low regulatory burden of obtaining these samples, they may be reasonable mechanical surrogates for brain tissue and tumors in certain testing applications.

**Fig 8:**
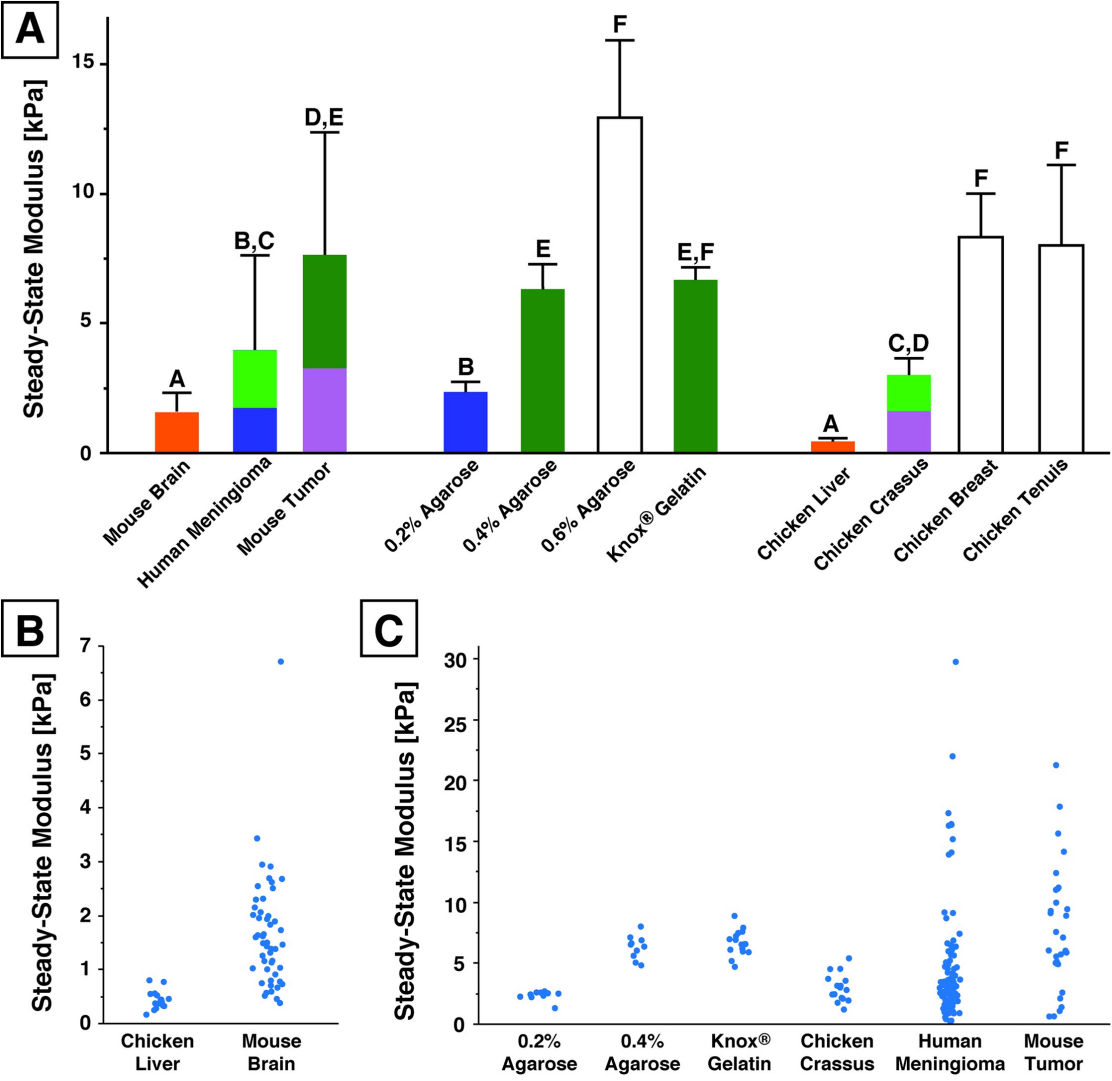
A wide variety of commercially available materials can serve as mechanical surrogates for human brain tissue and tumors. (A) SSM mean for each tissue (as an average of each sample mean) with the corresponding standard deviation; samples with the same letter and color are **not** statistically different from each other based on a multiple comparison Wilcoxon test (Table 1). (B) Readily obtainable chicken liver shows similar steady-state moduli to normal mouse brain. (C) The crassus muscle of the chicken gizzard is mechanically similar to both human meningiomas and mouse tumors, and non-living hydrogels can be fabricated with concentrations similar to tumors.

Since hydrogels commonly used as mechanical brain surrogates had higher SSM than normal brain, we hypothesized that a commercially available tissue may be able to better match the small range of brain tissue and tumor SSM. Of all commercial tissues tested, only chicken liver had an SSM similar to normal brain tissue (p = 0.4727) and lower than human meningiomas (p = 0.0006, Figs 7 and 8 and Table 1). Chicken liver from local grocers is also readily available, inexpensive, and typically unregulated for research purposes, so it may be a reasonable substitute for brain in select testing applications.

Human brain tumors are more complex to mimic mechanically, as is evidenced by their high variability. The crassus muscle of the chicken gizzard had a similar SSM of 3.00 ± 0.65 kPa to both human meningiomas (3.97 ± 3.66 kPa, p = 0.0969) and higher modulus mouse tumors (7.64 ± 4.73 kPa, p = 0.1427). Low concentration agarose hydrogels and Knox® gelatin were also similar to brain tumors, despite wide-spread citation as a surrogate for normal tissue [21,22].

**Fig 9:**
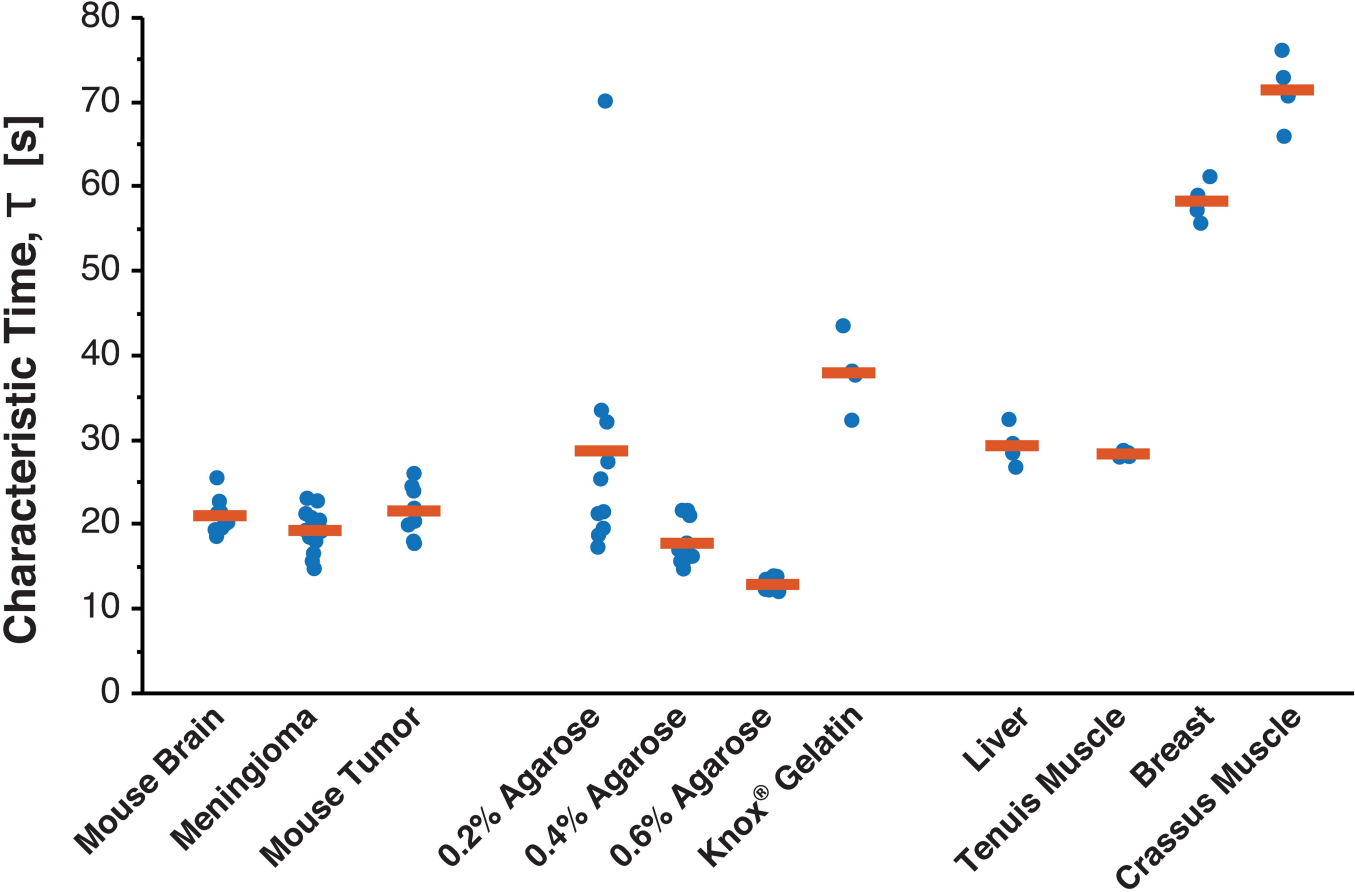
Characteristic time demonstrates similar viscous relaxation behavior of normal brain and brain tumors. Graph depicts the characteristic time, τ, of potential mechanical surrogates for brain tissue and tumors. τ is determined from fitting the calculated effective modulus (Eq 2) during stress relaxation to the SLS model of viscoelasticity (Eq 3). Similar relaxation behavior can be seen between potential surrogates, and based on recent studies, capturing the viscous behavior of biomimetic materials is equally important as elastic behavior.

Lastly, we analyzed characteristic time, τ, to compare the viscous relaxation of human brain tumors and potential mechanical surrogates (Fig 9). Most constitutive models of the brain require assumptions about viscosity [36], and it has been recently demonstrated that both viscous and elastic properties of hydrogels can regulate cell behavior [54].

While statistical significance varied, the characteristic time of human meningiomas (19.29 ± 2.20 s, mean ± std. dev.), murine brain tumors (21.57 ± 3.06 s), and murine brain (21.01 ± 2.00 s) were all similar in magnitude (Table 2). In comparison to potential surrogates, human meningiomas and 0.2% agarose hydrogels (28.71 ± 15.58 s) were significantly different in their characteristic time (p = 0.0135) in contrast to their similar SSM. Instead, human meningiomas had a similar characteristic time to 0.4% agarose hydrogels (17.75 ± 2.71 s, p = 0.1436). Human meningiomas also had lower characteristic times than chicken liver (29.32 ± 2.39 s, p = 0.0025) and crassus muscle (71.44 ± 4.28 s, p = 0.0025). These data reinforce the importance of selecting surrogates that match the physical properties with the most relevance to future experiments since it is often hard to match all physical properties of interest with one material.

**Table 2:** Results of multiple comparisons of characteristic time of potential surrogates.

*Wilcoxon p-values for Characteristic Decay Time*		Human	Mouse		Chicken				Agarose			Gelatin
		Menin^a^	Tumor	Brain	Liver	Crassus	Tenuis	Breast	0.2%	0.4%	0.6%	Knox®
**Human**	Menin^a^		0.1266	0.0466	0.0025	**0.0025**	0.0025	0.0025	**0.0135**	0.1436	<0.001	0.0025
**Mouse**	Tumor	0.1266		0.7558	0.0085	**0.0085**	0.0085	0.0085	0.3508	**0.0185**	<0.001	**0.0085**
	Brain	0.0466	0.7558		**0.0058**	0.0058	0.0058	0.0058	0.2413	0.0312	<0.001	0.0058
**Chicken**	Liver	0.0025	0.0085	**0.0058**		0.0304	0.6650	0.0304	0.2888	0.0058	0.0032	0.0606
	Crassus	**0.0025**	**0.0085**	0.0058	0.0304		0.0304	0.0304	0.0089	0.0058	0.0032	0.0304
	Tenuis	0.0025	0.0085	0.0058	0.6650	0.0304		0.0304	0.2888	0.0058	0.0032	0.0304
	Breast	0.0025	0.0085	0.0058	0.0304	0.0304	0.0304		0.0284	0.0058	0.0032	0.0304
**Agarose**	0.2%	**0.0135**	0.3508	0.2413	0.2888	0.0089	0.2888	0.0284		0.0073	<0.001	0.0403
	0.4%	0.1436	**0.0185**	0.0312	0.0058	0.0058	0.0058	0.0058	0.0073		<0.001	0.0058
	0.6%	<0.001	<0.001	<0.001	0.0032	0.0032	0.0032	0.0032	<0.001	<0.001		0.0032
**Gelatin**	Knox®	0.0025	**0.0085**	0.0058	0.0606	0.0304	0.0304	0.0304	0.0403	0.0058	0.0032	

P-values were obtained by performing a non-parametric Wilcoxon test for multiple comparisons in JMP software (SAS).

Grey boxes indicate tissues/surrogates that are *not* statistically different, and values in bold indicate potential surrogates identified considering elastic properties though time-dependent properties are dissimilar.

^a^Menin = human meningioma

We have identified mechanical surrogates that reasonably match the steady-state modulus of brain tissue and tumors, but additional off-the-shelf hydrogels and custom biomaterials can be tailored to create mechanical surrogates for tissue with additional desired properties. Hydrogels of varying stiffnesses and geometries could also be combined together to match the heterogeneity demonstrated in normal brain tissue [55,56] that is likely causing the high variability in brain tumor results in this study. However, if intra-sample heterogeneity is critical to one’s hypothesis or experiment, our techniques would not be the best to determine appropriate surrogates. Techniques such as computed tomography [22] and AFM may be more appropriate. Our methods also are inadequate to evaluate mimics for time-dependent constitutive behaviors relevant to computational models or blast injury, for example. As we demonstrate here, proposed surrogates should be carefully characterized using relevant methods to ensure applicability.

Though *in vitro* testing surrogates can never replicate freshly excised tissue for quasi-static mechanical tests, the comparison of SSM between potential surrogates can provide insight into target values for biomaterial design and device testing.

## Conclusion

With our custom indentation system, we quantified the steady-state modulus of many common mechanical brain surrogates as well as freshly isolated human brain tumors. We have directly quantified the mechanical properties of human brain tumors, and our 2–5 times higher modulus corresponds with published findings from other indirect characterization methods. We have shown that surrogates often used to mimic brain, such as agarose and ballistic gel, actually have higher steady-state moduli than normal brain tissue that may make them better mechanical testing surrogates for brain tumors. Common, inexpensive store-bought products such as poultry meat and gelatin have similar quasi-static properties to normal and diseased brain tissue, which provides easy and affordable access to mechanical surrogates of the brain for studies of device-tissue interactions. Of the tissues tested, chicken liver would seem to be a reasonable quasi-static mechanical surrogate for normal brain and the crassus muscle of the gizzard as a reasonable surrogate for primary tumors. Of the hydrogels tested, agarose could be tuned to mimic tumor tissue, and Knox® gelatin is also a reasonable surrogate for tumors. Similar organs from larger animals may be necessary for testing larger devices, however, and could be characterized for comparison as well. Careful characterization of widely available tissues and hydrogels and their comparison to hard-to-obtain clinical samples can help identify mechanical surrogates for a wide range of applications where patient samples are unobtainable or unreasonable to use for experimental work.

## Supporting information

S1 Fig Elastomers demonstrate no stress-relaxation behavior. Due to their viscoelastic properties, biological tissues and other soft matter often undergo stress relaxation in resonse to a constant, applied strain. To demonstrate that stress-relaxation observed in our brain tumor measurements was not the result of slippling or wetting of the surface, we indented a material known to be elastic for the given strains and time-frames. Sylgard 184 (Dow Corning) silicone pre-polymer base and curing agent were mixed at a 10:1 w:w ratio, poured into the bottom of a small petri dish, dessicated for 1 hour, and cured overnight in 50^°^C oven. The silicone was then indented using the same submerged methodology as was done for all other samples in the study.
(TIF)

S2 Fig Standard linear solid model fits well to experimentally calculated effective modulus. Effective modulus as a function of time (blue lines) is determined based on using a modified Hertz contact model (Eq 2). To determine the SSM, we fit the calculated effective modulus to the SLS model of viscoelasticity (Eq 3, red lines). To determine the goodness of the fit, the NMSE is calculated to verify that the SLS fit matches the experimental data. In most cases, NMSEs above 0.8 were observed. Even though lower NMSE values still seemed to be a reasonable fit, indentations with NMSE < 0.4 were excluded.
(TIF)

S3 Fig Mechanical properties of collagen hydrogels can be tuned to change SSM. The SSM of collagen-based hydrogels can be tuned via collagen concentration in solution and treatment with gluteraldehyde. Graph depicts the average SSM of each collagen gel tested at concentrations of 2, 3, and 4 mg/mL of collagen in solution alongside 0, 2, or 4 hour treatment post-gelation in gluteraldehyde solution.
(TIF)

S1 Table Raw data used to construct figures and tables. (XLSX)
