# Supplementary figures and images for: Mechanical characterization of human brain tumors from patients and comparison to potential surgical phantoms
Source: PLoS One. 2017 Jun 5;12(6):e0177561. doi: 10.1371/journal.pone.0177561 (PMC5459328; doi:10.1371/journal.pone.0177561)

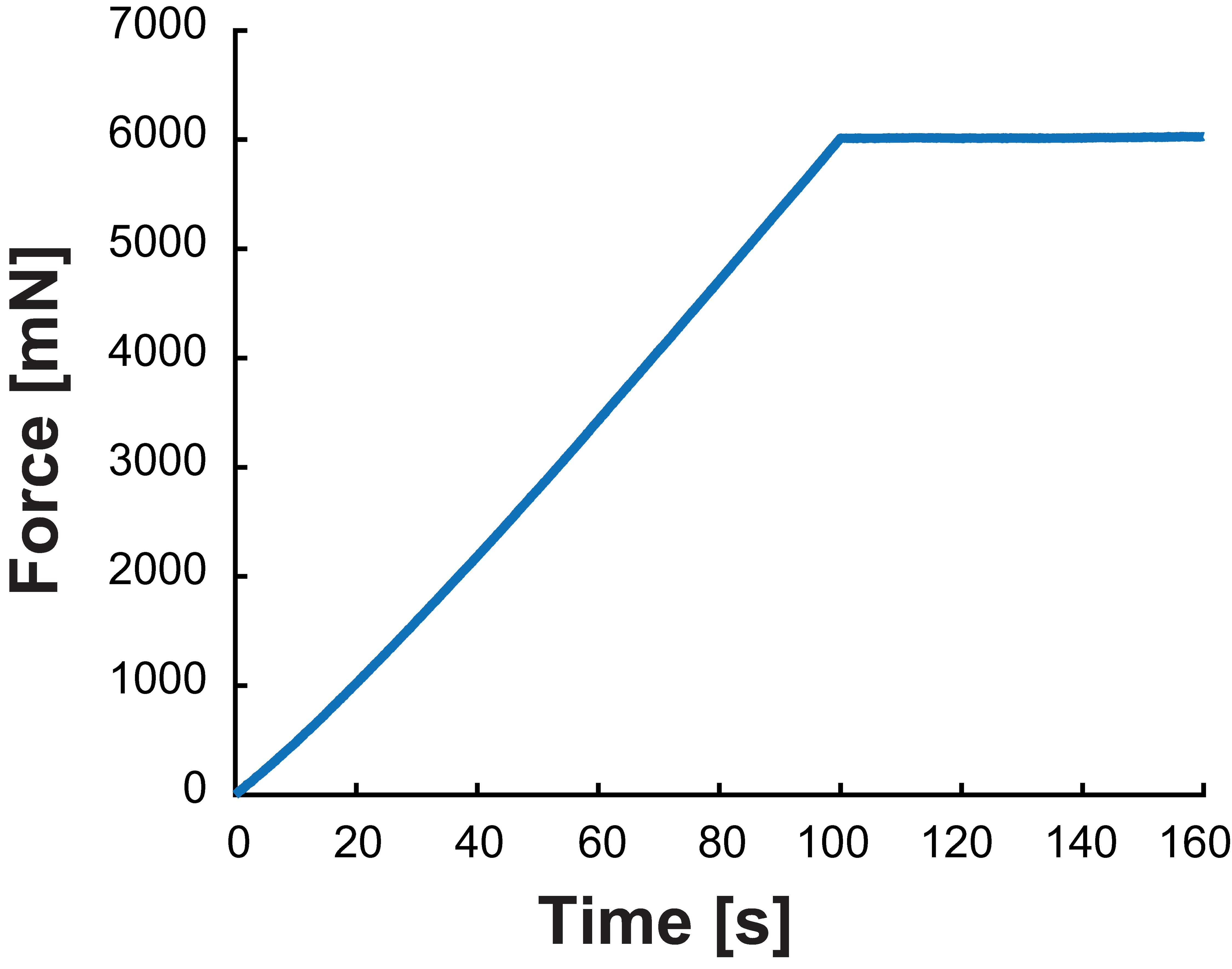

Supplement: S1 Fig — Due to their viscoelastic properties, biological tissues and other soft matter often undergo stress relaxation in resonse to a constant, applied strain. To demonstrate that stress-relaxation observed in our brain tumor measurements was not the result of slippling or wetting of the surface, we indented a material known to be elastic for the given strains and time-frames. Sylgard 184 (Dow Corning) silicone pre-polymer base and curing agent were mixed at a 10:1 w:w ratio, poured into the bottom of a small petri dish, dessicated for 1 hour, and cured overnight in 50°C oven. The silicone was then indented using the same submerged methodology as was done for all other samples in the study. (TIF) [file pone.0177561.s001.tif]

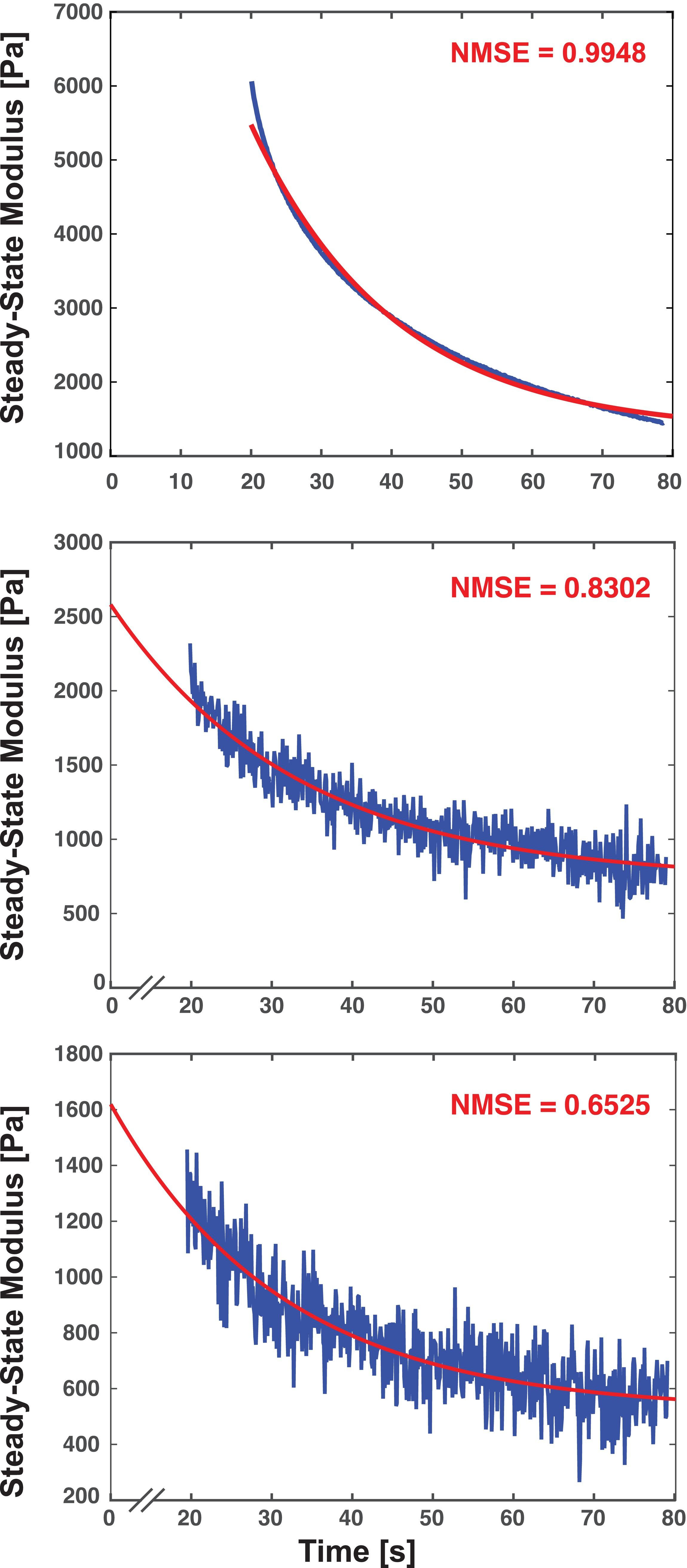

Supplement: S2 Fig — Effective modulus as a function of time (blue lines) is determined based on using a modified Hertz contact model (Eq 2). To determine the SSM, we fit the calculated effective modulus to the SLS model of viscoelasticity (Eq 3, red lines). To determine the goodness of the fit, the NMSE is calculated to verify that the SLS fit matches the experimental data. In most cases, NMSEs above 0.8 were observed. Even though lower NMSE values still seemed to be a reasonable fit, indentations with NMSE < 0.4 were excluded. (TIF) [file pone.0177561.s002.tif]

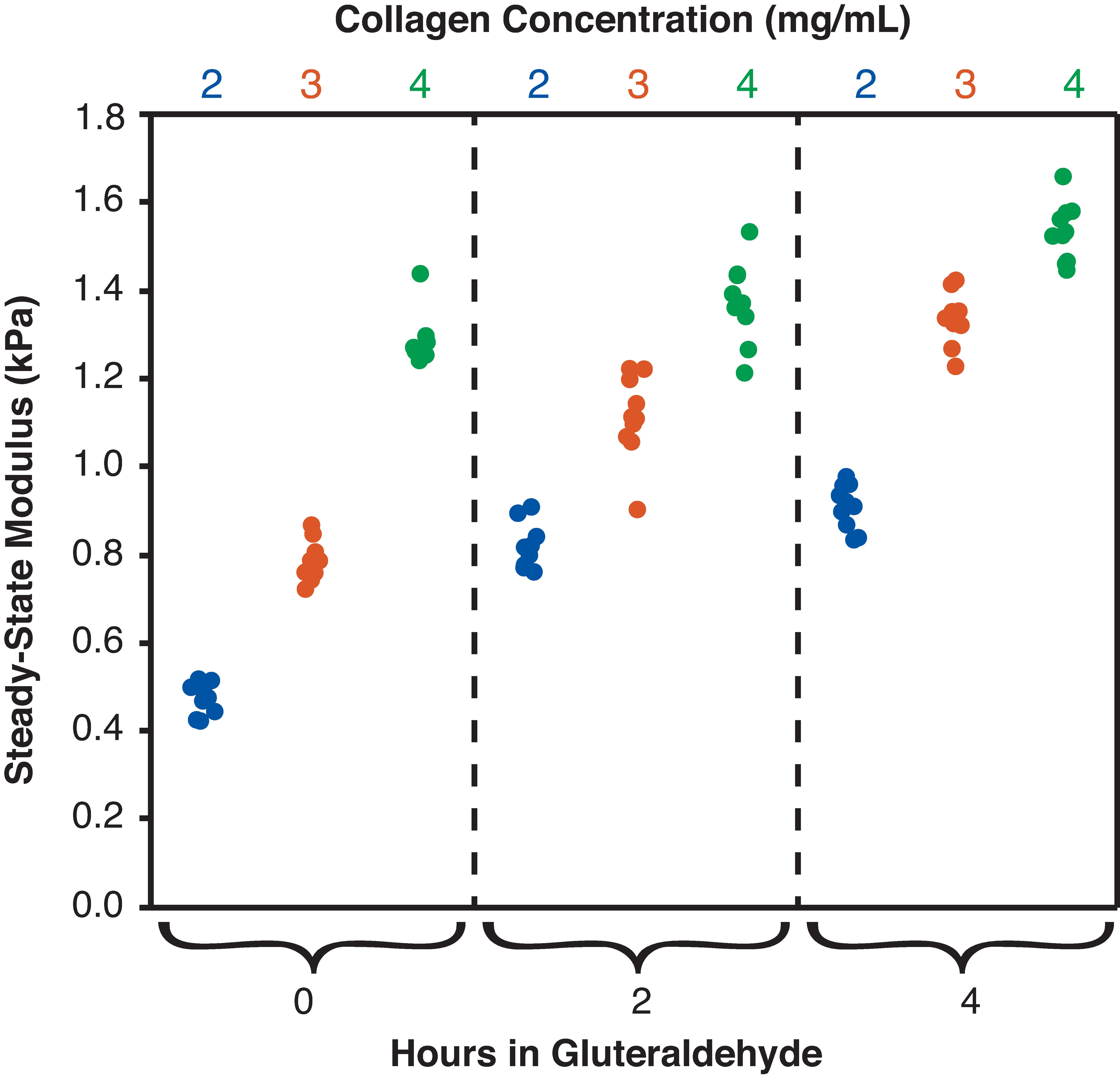

Supplement: S3 Fig — The SSM of collagen-based hydrogels can be tuned via collagen concentration in solution and treatment with gluteraldehyde. Graph depicts the average SSM of each collagen gel tested at concentrations of 2, 3, and 4 mg/mL of collagen in solution alongside 0, 2, or 4 hour treatment post-gelation in gluteraldehyde solution. (TIF) [file pone.0177561.s003.tif]
